# 

**Published:** 1846-07

**Authors:** 


### MEDICO-CHIRURGICAL

### REVIEW,

AND

### JOURNAL OF PRACTICAL MEDICINE.

NEW SERIES.

VOL. IV.

Nothing to index

APRIL 1, TO SEPTEMBER 31, 1846.

Surgeon Gen'l's Office LIBRARY 57465

LONDON:
SAMUEL HIGHLEY, 32, FLEET STREET,
1846.

WI
ME75T

PRINTED BY F. HAYDEN,
Little College Street, Westminster.

### BIBLIOGRAPHICAL RECORD.

1. A Manual of Physiology, including Physiological Anatomy, for the Use of the Medical Student. By *William B. Carpenter,* M.D., with one hundred and eighty illustrations. Foolscap 8vo, pp. 595. London, 1846.

2. Fragments of Medical Science and Art. An Address delivered before the Boylston Medical Society of Harvard University. By *Henry Jacob Bigelow,* M.D. 8vo, pp. 54. Boston.

*A masterly exposition of the principles of the Baconian philosophy as applied to medical science. It is written with great power of reasoning, and its language is excellent. It deserves to be re-printed in this country.*

3. The Medical Police of the United Kingdom. From the Westminster Review for March, 1846. 8vo, pp. 35.

*An able article, that deserves to be generally known.*

4. The Monthly Journal of Medical Science. Edited by *John Rose Cormack,* M.D. April to June, 1846. Edinburgh.

5. Scrofula, its Nature, its Causes, its Prevalence, and the Principles of Treatment. By *Benjamin Phillips.* Illustrated with an engraved plate. 8vo, pp. 379. Lonnon, 1846.

6. The Young Stethoscopist, or the Student's Aid to Auscultation. By *Henry J. Bowditch,* M.D. Foolscap 8vo, pp. 285. Boston.

7. Memoranda on Difficult Subjects in Anatomy, Surgery, and Physiology, forming a Pocket Companion for the Young Surgeon, or for Students preparing for Examinations. 2nd Edition, re-compiled and enlarged by *Mark Noble Bower.* 18mo, pp. 276. London.

*Multum in parvo.*

8. An Atlas of Anatomical Plates of the Human Body, accompanied with Descriptions in Hindustani. By *Frederic J. Mouat,* M.D., F.R.C.S.E., &c., Assisted by Moonsshi Nusseerudin A hmud, late of the Calcutta Madrussa. Fasciculus I., containing the Bones. Folio. Calcutta, 1846.

*Very great praise is due to Dr. Mouat,—who is one of the professors in the Bengal Medical College, at which there are 150 native students regularly engaged in dissection!—for this publication. The drawings on stone were made by Mr. Grant; and the letter-press has been selected from the writings of Quain, Meckel, and Bell. Four other fasciculi, to complete the work, are promised,*

9. Chelius' System of Surgery. Translated by *South.* Part II.

10. Elements of the Theory and Practice of Medicine. By *George Gregory,* M.D., Fellow of the Royal College of Physicians, Physician to the Small-pox Hospital, &c. Sixth edition, 8vo, pp. 805. 1846.

*By avoiding all minute details on unimportant matters, and carefully separating the wheat from the chaff, Dr. G. has compressed into this edition —the sixth in the course of 25 years—a very large amount of useful information. No safer guide can be put into the hands of the young practitioner. It is eminently practical.*

11. Remarks on the Dysentery and Hepatitis of India. By *E.A. Parkes,* M.B., late Assistant Surgeon H.M. 84th Regt. 8vo, pp. 283. 1846.

*In our next.*

12. On the Health of Towns as influenced by defective Cleansing and Drainage, and on the Applicatiou of the Refuse of Towns to Agricultural Purposes. By *W. A. Guy,* M.B. 8vo, pp. 48. 1846.

13. The Mineral Waters of Kreuznach. By *J. E. P. Prieger,* M.D., Principal Physician of the Royal Hospital at Kreuznach. 8vo, pp. 104. 1846.

14. Manual of Operative Surgery, based on Normal and Pathological Anatomy. By *J. F. Malgaigne.* Translated from the French, by *F. Brittain,* A.B., M.D., &c. Foolscap 8vo, pp. 602. Woodcuts. 1846.

*In our next.*

15. On Disorders of the Cerebral Circulation, and on the Connexion between Affections of the Brain and Diseases of the Heart. By *George Burrows,* M.D., Fellow of the Royal College of Physicians, London, Physician and Lecturer on Medicine at St. Bartholomew's Hospital. 8vo, pp. 236. Coloured plates. 1846.

16. The "Open Surgery" Question considered, and its General Adoption advocated. By *W. J. Preston,* Surgeon and Apothecary. 8vo, pp. 25. 1846.

17. Lectures on Urine, and on the Pathology, Diagnosis, and Treatment of Urinary Diseases. By *J. Aldridge,* MD., Lecturer on Chemistry at the Medical School, Park-street, Dublin. 8vo, pp. 80. 1846.

*These Lectures give an excellent summary of the leading facts and discoveries touching the pathology of the urine. To the man engaged in active practice, we strongly recommend their attentive perusal.*

18. Meade's Manual for Students preparing for Examination at Apothecaries' Hall and other Medical Institutions. Second Edition, improved and corrected. Foolscap 8vo, pp. 544. 1846.

*The first edition was published in* 1839. *As a compendium of the leading facts in chemistry, materia medica and toxicology, anatomy, practice of medicine, &c., it is one of the best manuals which the student can consult.*

19. The Northern Journal of Medicine. Edited by *William Seller*, M.D. April, May, and June.

20. A Practical Treatise on the Diseases of Children. By *James M. Coley*, M.D. 8vo, pp. 480. London, 1846.

*In our next.*

21. Commentary on the Hindoo System of Medicine. By *T. A. Wise*, M.D. Bengal Medical Service. 8vo, pp. 451. Calcutta. 1845.

*In our next.*

22. Clinical Illustrations of the Diseases of India, as exhibited in the Medical History of a Body of European Soldiery for a Series of Years from their arrival in that Country. By *William Geddes*, M.D., &c. 8vo, pp. 502. London, 1846.

*In our next.*

23. Introductory Lectures to a Course of Military Surgery, delivered in the University of Edinburgh. By Sir *George Ballingall.* Royal 8vo, pp. 33. Edinburgh, 1846.

*A very interesting report of Sir George's recent visit to the hospitals on the Continent, in Malta, Egypt, &c. It is written with excellent good-feeling, and a hearty love of his subject.*

24. Deontologie Medicale ou des Devoirs et des Droits des Medecine. Par le Docteur *Max Simon.* 8vo, pp. 567. Paris, 1845.

*In our next.*

25. A Practical Manual, containing a Description of the General, Chemical, and Microscopical Characters of the Blood and Secretions of the Human Body, as well as of their Components, including both their healthy and diseased states; with the best Method of separating and estimating their Ingrediency. Also, a succinct Account of the various Concretions occasionally found in the Body, and forming Calculi. By *John William Griffiths*, M.D. 8vo, pp. 176. London, 1846. *In our next.*

26. Outlines of the Nerves, with short Descriptions, designed for Medical Students. By *John Neill*, M.D. Royal 8vo, pp. 30. Philadelphia, 1845.

27. Outlines of the Arteries, with short Descriptions, designed for Medical Students. By *John Neill*, M.D. Royal 8vo, pp. 30. Philadelphia, 1845.

28. On the Treatment of Strictures of the Urethra by Mechanical Dilatation, and other Diseases attendant on them; with some Anatomical Observations on the Natural Form and Dimensions of the Urethra, with a view to a more precise adaptation and use of the Instruments employed in their Relief. By *James Briggs*, Senior Surgeon to the Lock Hospital, &c. 8vo, pp. 62, Sewed. London, 1845.

29. Notes and Recollections of a Professional Life. By the late *William Fergusson*, M,D., Inspector-General of Military Hospitals. Edited by his Son, *James Fergusson.* 8vo, pp. 264. London, 1846.

30. The Structure and Functions of the Female Breast, as they relate to its Health, Derangement, and Disease. By *W. Tuson*, F.R.S. FLS., Surgeon to the Middlesex Hospital. 8vo, pp. 512. London, 1846.

31. Remarks upon Medical Organization and Reform (Foreign and English). By *Edwin Lee*, Fellow of the Royal Medical and Chirurgical Society, &c. 8vo, pp. 128. London, 1846.

32. Hospitals for Troops in the West Indies. By *Hugh Bone*, M.D. 8vo, pp. 85. Edinburgh, 1846.

33. The American Journal of the Medical Sciences. Edited by *Isaac Hays*, M.D. Jan. and April, 1846. Philadelphia.

24. Liebig's Physiology, applied in the Treatment of Functional Derangement and Organic Disease, with Observations on Hahnemann's Practice. Part I. By *John Leeson.* 8vo, pp. 223. London, 1846. *In our next.*

25. The Elements of Pathological Anatomy. Illustrated by coloured Engravings, and 250 Woodcuts. By *Samuel D. Gross*, M.D. Second Edition, thoroughly revised and greatly enarged. Royal 8vo, pp. 822. Philadelphia, 1845.

26. Three Reports by the Joint Deputation of the Society of Apothecaries and the National Association of General Practitioners, appointed to confer with the State on the subject of the Incorporation of the General Practioners, in Medicine, Surgery, and Midwifery. 8vo, pp. 54. London, 1846.

27. Historical and Critical Remarks on the Operations for the Cure of Cataract. By *Alex. Watson*, M.D. (From the Edinburgh Medical and Surgical Journal, No. 165.) 8vo, pp. 35. Edinburgh, 1845.

*Elaborate and ably written.*

28. Inaugural Dissertation on Yellow Fever, and on the Treatment of that Disease by Saline Medicines. By *George F. Bone*, M.D. With an Appendix, On the principles to be observed in providing Barracks, &c.

29. Outlines of the Course of Qualitative Analysis followed in the Giessen Laboratory. By *Henry Will*, Ph. D. With a Preface by *Baron Liebig.* 8vo, pp. 114. London, 1846.

30. A Series of Essays on Inflammation and its Varieties, tending to show that most Diseases either consist in Inflammation, or are consequences of it more or less remote. Essay 1. The Natural History of the Disease, with Preliminary Observations. By *Henry Clutterbuck*, M.D. 8vo, pp. 95. London, 1846.

31. Observations on the Edinburgh Pharmacopœia, and on the Dispensatories of Dr. *Christison* and Dr. *A. T. Thompson:* to which are added, Illustrations of the present State of Pharmacy in England. By *Richard Phillips*, F.R.S. L. & E., and Honorary Member of the Pharmaceutical Society. 8vo, pp. 94. London, 1846.

*These Observations deserve the attentive perusal of the pharmaceutical chemist. Part of them appeared in our numbers for January and April,* 1845.

32. Hand-Book of Anatomy for Students of the Fine Arts, containing a Description, with Woodcut Illustrations, of the Skeleton and External Muscles of the Human Figure. By *J. A. Wheeler*. New Edition, foolscap 8vo.

*This little work, consisting of well-executed woodcuts of the bones and muscles, will prove a useful portable companion for artists.*

33. Practical Surgery. By *Robert Liston*, Fourth Ediition, 8vo, pp. 590. London, 1846.

*In our next.*

